# Perioperative Predictive Factors for Positive Outcomes in Spine Fusion for Adult Deformity Correction

**DOI:** 10.3390/jcm11010144

**Published:** 2021-12-28

**Authors:** Alice Baroncini, Filippo Migliorini, Francesco Langella, Paolo Barletta, Per Trobisch, Riccardo Cecchinato, Marco Damilano, Emanuele Quarto, Claudio Lamartina, Pedro Berjano

**Affiliations:** 1 IRCCS Istituto Ortopedico Galeazzi, Via Riccardo Galeazzi, 4, 20161 Milan, Italy; francesco.langella.md@gmail.com (F.L.); paolo.barletta@grupposandonato.it (P.B.); dott.cecchinato@gmail.com (R.C.); marco.damilano@gmail.com (M.D.); emanuelequarto88@gmail.com (E.Q.); c.lamartina@chirurgiavertebrale.net (C.L.); pberjano@gmail.com (P.B.); 2 Department of Orthopaedic Surgery, RWTH Uniklinik Aachen, 52074 Aachen, Germany; fmigliorini@ukaachen.de; 3 Department of Spine Surgery, Eifelklinik St. Brigida, 52152 Simmerath, Germany; per.trobisch@artemed.de

**Keywords:** adult spine deformity, adult spine fusion, deformity correction, perioperative parameters, ODI, VAS, disability

## Abstract

Purpose: Identifying perioperative factors that may influence the outcomes of long spine fusion for the treatment of adult deformity is key for tailored surgical planning and targeted informed consent. The aim of this study was to analyze the association between demographic or perioperative factors and clinical outcomes 2 years after long spine fusion for the treatment of adult deformity. Methods: This study is a multivariate analysis of retrospectively collected data. All patients who underwent long fusion of the lumbar spine for adult spinal deformity (January 2016–June 2019) were included. The outcomes of interest were the Oswestry disability index (ODI), visual analogic scale (VAS) preoperatively and at 1 and 2 years’ follow up, age, body mass index, American Society of Anaesthesiologists (ASA) score, upper and lowest instrumented vertebrae (UIV and LIV, respectively), length of surgery, estimated blood loss, and length of hospital stay. Results: Data from 192 patients were available. The ODI at 2 years correlated weakly to moderately with age (*r =* 0.4), BMI (*r =* 0.2), ASA (*r =* 0.3), and LIV (*r =* 0.2), and strongly with preoperative ODI (*r =* 0.6). The leg VAS at 2 years moderately correlated with age (*r =* 0.3) and BMI (*r =* 0.3). Conclusion: ODI and VAS at 2 years’ follow-up had no to little association to preoperative age, health status, LIV, or other peroperative data, but showed a strong correlation with preoperative ODI and pain level.

## 1. Introduction

The social burden caused by low back pain (LBP) is relevant, having a first-ever episode incidence of 15% and an 80% recurrence rate within a year [1]. This percentage increases in patients affected by adult spine deformity [2] and various studies showed that this condition has a negative impact on the patients’ quality of life [3,4]. Surgical deformity correction involves complex procedures; given the advances in surgical and anesthesiological techniques, it is now possible to perform surgery in patients at an older age and with more comorbidities [5,6,7,8]. So, disability and pain levels play a decisive role in the assessment of a patient and in the decision-making process [9]. However, the postoperative motion restriction following fusion of the lumbar spine should be considered when indicating surgical management to ensure that the benefits of the surgery outweigh the limitations [10].

Patient-reported outcome measures (PROMs) are used to obtain a more complete overview of a patient’s status, as they allow to match objective informations such as radiographic findings with subjective data regarding different aspects of the patient’s quality of life [11]. In particular, the Oswestry disability index (ODI) and the visual analogic scale (VAS) are two parameters widely used for pre- and postoperative assessment of patients undergoing spine surgery [12,13]. 

The effects of the correction of sagittal and coronal parameters on disability and pain levels have been evaluated in multiple studies [14,15,16,17]. However, the effects of demographic and perioperative data on the postoperative outcome has not yet been thoroughly investigated, and patients with a low risk of a poor clinical outcome have not yet been characterized [18]. Thus, the aim of this study was to analyze the demographic and perioperative data of adult spine deformity patients undergoing long fusion involving the lumbar spine, in order to seek possible associations between these parameters and levels of disability (ODI) and pain (VAS back and leg) at the one- and two-year follow-up.

## 2. Materials and Methods

### 2.1. Patient Recruitment

The present retrospective study was conducted according to the Strengthening the Reporting of Observational Studies in Epidemiology: the STROBE Statement [19].

All patients who underwent spine fusion at IRCCS Istituto Ortopedico Galeazzi (Milano, Italy) between January 2016 and June 2019 were retrospectively screened for inclusion on the local spine registry using the ICD (International Classification of Diseases) diagnosis and procedure codes listed in Table 1. The use of ICD codes for diagnosis and procedure allows to retrieve data from the registry, but also offers an internationally acknowledged key to replicate data extraction, if necessary. Inclusion criteria for the current study were age ≥ 18, diagnosis of adult spine deformity, and fusion of at least four segments—at least three of which in the lumbar spine. Patients who did not have an ODI and/or VAS preoperatively and at the one- or two-year follow-up were not eligible for the study.

### 2.2. Outcomes of Interest

We analyzed the effects of demographic and perioperative parameters on ODI and VAS over time, as well as the mutual association between ODI and VAS at different follow-ups. Furthermore, question n. 11 of the COME back questionnaire (CB11) [20] was used to identify whether patients felt overall that surgery had helped or not (0 = helped a lot, 4 = made things worse). Demographic parameters included age, sex, body mass index (BMI), and American Society of Anaesthesiologists (ASA) score. The level of the upper and lowest instrumented vertebra (UIV and LIV, respectively) was analyzed. Length of surgery, estimated blood loss (EBL), and length of hospital stay were also considered.

### 2.3. Statistical Analysis

For the statistical analysis, STATA software (StataCorp, College Station, TX, USA) was used. Continuous variables are expressed as mean ± standard deviation. Comparisons between continuous variables across the follow-ups were assessed through the mean difference and t-test, with values of *p* < 0.05 considered statistically significant. A multivariate diagnostic through the Pearson product-moment correlation coefficient (*r*) was performed to investigate potential correlations between continuous variables. According to the Cauchy–Schwarz equation of inequality, the final effect can score between +1 (positive linear correlation) and −1 (negative linear correlation). Values of 0.1 > |*r*| < 0.3, 0.3 < |*r*| < 0.5, and |*r*| > 0.5 indicate weak, moderate, and strong association, respectively. The test of overall significance was performed through the χ2 test, with values of *p* > 0.05 considered statistically significant. 

## 3. Results

### 3.1. Patient Recruitment and Demographics

**Figure 1:**
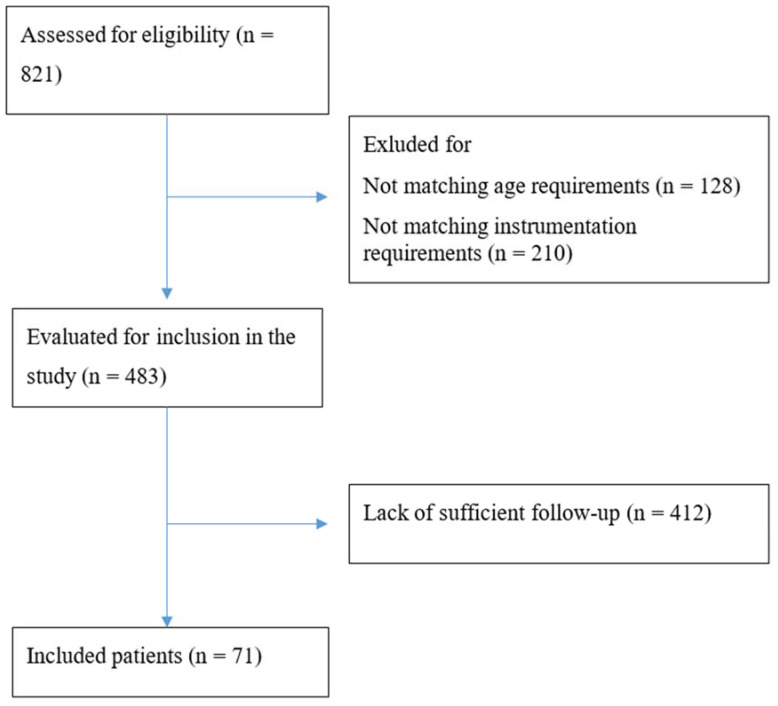
STROBE flow diagram of patient selection.

After cross-referencing the ICD diagnosis and procedure codes, 821 eligible patients were identified on the local spine registry. Of them, 128 were excluded because they were <18 years old. A further 210 were excluded because their level or extent of instrumentation did not match the requirements of this study. A further 291 patients were excluded due to the lack of a sufficient follow-up, leaving 192 patients available for the analysis. The flowchart of the patients’ recruitment is presented in Figure 1.

Summaries of the patients’ demographics and the considered intraoperative data are shown in Table 2 and Table 3, respectively. An overview of ODI, VAS, and CB11 in the different follow-ups is presented in Table 4. 

### 3.2. Multivariate Analysis

Age and BMI showed a significant, weak-to-moderate correlation with most of the considered PROMs (ODI and leg VAS before and after surgery, and back VAS preoperatively and at 12 months, CB11). The ASA class correlated moderately with the ODI at all follow-ups and with the VAS leg before surgery and at 1 year, and with the CB11 at both follow-ups. Length of surgery, EBL, and length of hospital stay had a little correlation to ODI, VAS, and CB11 at different follow-ups. While UIV showed no significant correlation with postoperative outcomes, LIV had a weak-to-moderate correlation with postoperative ODI, leg VAS, and CB11. Numerous, mostly medium-to-strong correlations were observed among ODI, leg and back VAS, and CB11. 

**Figure 2:**
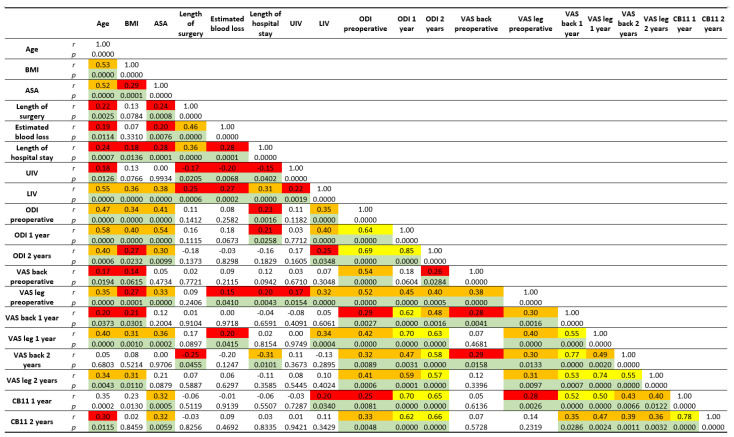
Overview of all the observed correlations among the considered parameters. Red, orange and yellow color indicate significant weak, moderate and strong correlations, respectively.

Other moderate correlations of interest were observed between age and BMI (*r* = 0.52, *p* < 0.001), ASA (*r* = 0.51, *p* < 0.001), and LIV (*r* = 0.54, *p* < 0.001); and between LIV and BMI (*r* = 0.35, *p* < 0.001), ASA (*r* = 0.38, *p* < 0.001), and length of hospital stay (*r* = 0.31, *p* < 0.001). Length of surgery correlated with EBL (*r* = 0.46, *p* < 0.001) and length of hospital stay (*r* = 0.33, *p* < 0.001). The details of the correlations are shown in Figure 2.

## 4. Discussion

Overall, we observed a significant improvement in ODI and leg and back VAS at the last follow-up. CB11 analysis highlighted a high level of satisfaction after surgery, confirming the results of previous studies, which reported positive outcomes after surgical therapy for adult spine deformity [21,22].

The correlation between ODI and age, BMI, or ASA was moderate at the one-year follow-up, but the strength of these correlations was reduced at the two-year follow-up. The correlation between leg and back VAS and age, BMI, and ASA showed similar trends to those observed for the ODI: back pain weakly correlated with age and BMI before surgery and at the 1 year follow-up, but no significant correlation was observed at 2 years, or with ASA at any follow-up. Leg pain showed a weak-to-moderate correlation with all parameters and at all follow-up, except with ASA at the last follow-up. Similar trends were also observed for CB11. These data confirmed that older age and poorer overall health condition may have a moderate negative impact on the level of complications and disability or pain after surgery [23,24,25], but this negative influence dissipates over time. Thus, these patients can also expect positive outcomes after long spine fusion [26,27,28,29], but have to be adequately informed that a poorer preoperative health status correlates with longer recovery time. Surgeons, however, need to consider that obesity and age or comorbidities have a relevant impact on intraoperative blood loss, length of surgery, and complication rate; thus, preoperative BMI and ASA should still be considered when planning long spine fusion [30,31,32]. 

Length of surgery, estimated blood loss, and length of hospital stay showed no or only weak correlation with ODI, VAS, and CB11. This aspect is also key for the informed consent of the patients and their attitude toward the recovery process, as a prolonged hospital stay does not have a negative impact on the long-term outcomes of surgery. 

Analyzing the correlation of ODI, VAS, or CB with the extent of the instrumentation, we found that the level of the UIV did not affect any of the outcomes of interest. Given the relative limited mobility of the thoracic spine [33], these data are not surprising. It is however striking that the moderate correlation between ODI and LIV at the one-year follow-up was further reduced at the two-year follow-up. Similar results were obtained in other studies observing different PROMs and the ability of patients to perform determined activities after spinal fusion: over time, a gradual ODI improvement could be observed even in patients with fusion to the pelvis [10,34]. The explanation for this finding may lie in the postoperative movement restrictions required by many surgeons after fusion (e.g., avoiding forward bending or heavy lifting), which then ease over time, or in the fact that patients adapt to the movement restrictions imposed by the instrumentation and develop strategies to overcome them. This topic requires further investigation: if the developing of these strategies is the key in reducing postoperative disability after spine fusion, specific pre- or postoperative physiotherapy programs may be implemented to support patients and improve their quality of life after surgery. 

Overall, the ODI, VAS, and CB parameters showed multiple moderate and strong correlations amongst each other, confirming how different aspects of a patient’s health, quality of life, and satisfaction regarding treatment are interconnected [35]. Regarding the ODI, a strong correlation was observed between pre- and postoperative disability levels; this suggests that patients starting with high ODI values have lower chances of achieving a low ODI postoperatively. This represents a key factor in planning the timing of surgery. Different to what was observed for the ODI, the preoperative VAS only weakly to moderately associated with levels of back and pain level at the two-year follow-up. Thus, even patients with a high preoperative pain level can expect an improvement with respect to the painful symptoms two years after surgery. Unsurprisingly, the level of satisfaction with the treatment (CB11) correlated with ODI and VAS both at the one- and two-year follow-ups. However, while the correlation with pain level was of moderate intensity and declined at the two-year follow-up, the correlation to disability was strong at both follow-ups. A similar correlation between patients’ satisfaction and PROMs was also observed by another study group [35].

This study is not without limitations, the main one being its retrospective nature. The relationship between ODI, pain, and satisfaction with treatment and pre- and perioperative data proved to be a complex, and further research on a wider patient cohort will be required to investigate it. Furthermore, the patients in our cohort presented different types of instrumentations (e.g., different types or levels of interbody implants) and deformity correction techniques. While it was not possible to investigate the effect of different surgical techniques on the outcome of interest due to the limited number of observations, this topic deserves further analysis in the future.

## 5. Conclusions

The main finding of this work was that preoperative ODI showed the strongest association with the postoperative clinical outcomes after spine fusion for adult deformity correction. Other parameters such as age, health status, or LIV presented only a weak association with the long-term ODI or VAS values. Thus, surgery should be performed in a timely manner to avoid patients reaching high preoperative ODI values. 

## Figures and Tables

**Table 1:** List of all ICD diagnosis and procedure codes used for data extraction from the local spine registry.

**ICD Diagnosis Codes**	
737.30, 737.31, 737.32, 737.34, 737.0, 737.10, 737.12, 737.22, 737.40, 737.41, 737.43, 737.19, 738.5, 737.39	
**ICD Procedure Codes**	
Primary surgery	81.05, 81.06, 81.08, 81.63, 81.64
Revision surgery	996.49, V45.4, 996.78, 998.89

ICD, International Classification of Diseases.

**Table 2:** Overview of the patients’ demographics.

Demographic Data	
Age (years)	53.4 ± 16.7
Sex	149 women (78%), 43 men (22%)
BMI (kg/cm^2^)	24.2 ± 3.9

BMI, body mass index.

**Table 3:** Summary of perioperative data.

Perioperative Data	
UIV	C7: 1; T1: 5; T2: 7; T3: 28; T4: 29; T5: 15; T6: 4; T7: 3; T8: 11; T9: 16; T10: 44; T11: 5; T12: 3; L1: 5; L2: 13; L3: 3
LIV	L3: 11; L4: 35; L5: 24; S1: 62; Ilium: 60
Access	Posterior only: 192; postero-anterior: 21; postero-lateral: 38
Curve correction method	SPO: 21; PSO: 13; ALIF: 21; LLIF 38
Length of surgery (min)	430 ± 150
% EBL	18 ± 15.3
EBL (mL)	1264 ± 1073
Length of hospital stay (days)	8.5 ± 4.5

UIV, upper instrumented vertebra; LIV, lowest instrumented vertebra; SPO, Smith Petersen osteotomy; PSO, pedicle substraction osteotomy; ALIF, anterior lumbar interbody fusion; LLIF, latera lumbar interbody fusion; EBL, estimated blood loss.

**Table 4:** Overview of ODI, back and leg VAS, and CB11 values over time.

ODI, VAS and CB11 Overview				
	Preop	1-year FU	2-years FU	*p* (Preop vs. 2-year FU)
ODI	42.5 ± 20.3	26.7 ± 21.4	26.8 ± 20.7	<0.0001
VAS back	6.8 ± 2.7	3.8 ± 3	4 ± 3.1	<0.0001
VAS leg	4.8 ± 3.7	3.4 ± 3.1	3.6 ± 3.5	0.01
CB11	-	0.9 ± 1.1	0.9 ± 1.2	-

ODI, Oswestry disability index; VAS, visual analogic scale; CB11, question n. 11 of the COME back questionnaire; FU, follow-up.

## Data Availability

The dataset used and/or analyzed in the present study is available from the corresponding author upon reasonable request.
